# Synthesis, Molecular Properties Prediction, and Anti-staphylococcal Activity of *N*-Acylhydrazones and New 1,3,4-Oxadiazole Derivatives

**DOI:** 10.3390/molecules17055095

**Published:** 2012-05-03

**Authors:** Cledualdo Soares de Oliveira, Bruno Freitas Lira, Vivyanne dos Santos Falcão-Silva, Jose Pinto Siqueira-Junior, Jose Maria Barbosa-Filho, Petronio Filgueiras de Athayde-Filho

**Affiliations:** 1 Department of Chemistry, Federal University of Paraiba, João Pessoa, PB 58059-900, Brazil; E-Mails: aldoscarchi@yahoo.com.br (C.S.O.); brunofrlira@hotmail.com (B.F.L.); 2 Department of Molecular Biology, Federal University of Paraíba, João Pessoa, PB 58059-900, Brazil; E-Mails: vivyannefalcao@yahoo.com.br (V.S.F.-S.); jpsiq@uol.com.br (J.P.S.-J.); 3 Laboratory of Pharmaceutical Technology, Federal University of Paraíba, João Pessoa, PB 58051-900, Brazil; E-Mail: jbarbosa@ltf.ufpb.br

**Keywords:** 1,3,4-oxadiazole, *N*-acylhydrazone, *Staphylococcus aureus*, drug-likeness

## Abstract

Five new 1-(2-(5-nitrofuran-2-yl)-5-(aryl)-1,3,4-oxadiazol-3-(2*H*)-yl) ethanone compounds **5a–e** were synthesized by cyclization of *N*-acylhydrazones **4a–e** with acetic anhydride under reflux conditions. Their structures were fully characterized by IR, ^1^H-NMR, and ^13^C-NMR. Furthermore, evaluations of the antibacterial activity of the 1,3,4-oxadiazoles **5a–e** and *N*-acylhydrazones **4a–e** showed strong activity against several strains of *Staphylococcus aureus*, with MICs between 4 μg/mL to 32 μg/mL. *In silico* studies of the parameters of Lipinski’s Rule of Five, as well as the topological polar surface area (TPSA), absorption percentage (% ABS), drug likeness and drug score indicate that these compounds, especially **4a** and **5d**, have potential to be new drug candidates.

## 1. Introduction

The introduction of antibiotics in the chemotherapy of bacterial infections in the middle of the last century revolutionized medicine, causing a drastic reduction in mortality from bacterial diseases. However, the spread and misuse of antibiotics has unfortunately helped the emergence of bacterial resistance, whereby bacteria populations have developed defense mechanisms against most antibiotics [1,2,3].

*Staphylococcus aureus* is often cited as the principal hospital pathogen that is becoming increasingly virulent and resistant to antibiotics, and the primary disseminator of human infectious diseases around the World [4]. The ease with which *S. aureus* acquires resistance to virtually all antibiotics currently in use is a matter of considerable concern [5,6].

**Figure 1:**
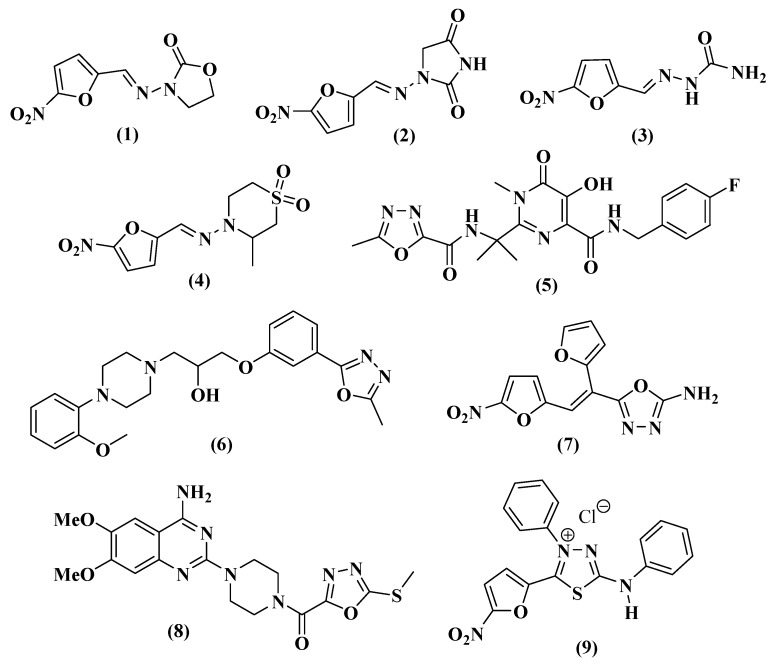
Chemical structures of biologically active molecules containing the 5-nitrofuran groups and/or 1,3,4-oxadiazole: furazolidone (**1**), nitrofurantoin (**2**), nitrofurazone (**3**), nifurtimox (**4**), raltegravir (**5**), nesapidil (**6**), furamisole (**7**), tiodazosin (**8**) and MC-2 (**9**).

Among compounds that possess a broad spectrum of biological activities are the nitrofurans. These compounds were the first nitroheterocyclic used in chemotherapy. Three of them, furazolidone (**1**), nitrofurantoin (**2**) and nitrofurazone (**3**) have been used to treat bacterial infections for more than 50 years [7,8]. Some derivatives of 5-nitrofuran, besides having antibacterial and antifungal properties, exhibit antiprotozoal activity as is the case of nifurtimox (**4**), used in the treatment of trypanosomiasis, and leishmaniasis [9,10]. Recently, it has also been reported that heterocyclic mesoionic compounds (**9**) containing the 5-nitrofuran group modulate bacterial resistance, putatively acting as efflux pump inhibitors [11], (Figure 1).

Another important group of heterocyclic compounds for medicinal chemistry are the 1,3,4-oxadiazoles which have been extensively reported in the literature for the synthesis of new biologically active molecules with antibacterial [12,13,14,15], antifungal [16,17], analgesic [18], anti-inflammatory [19], antiviral [20,21], antitumor [22,23], antihypertensive [24], enzyme inhibition [25,26] and anticonvulsant [27,28] activities. Examples of drugs containing the 1,3,4-oxadiazole unit currently used in clinical medicine are: raltegravir^®^ (**5**), an antiretroviral drug [29], nesapidil^®^ (**6**) an anti-arrhythmic therapy [30], furamizole^®^ (**7**) a nitrofuran derivative that has strong antibacterial activity [31] and tiodazosin^®^ (**8**) an antihypertensive drug [32] (Figure 1).

Therefore, considering that the 5-nitrofuran and 1,3,4-oxadiazole are important building blocks for the development of new drug candidates, we synthesized the intermediate *N*-acylhydrazones **4a–e** and the target 1,3,4-oxadiazoline molecules **5a–e** in order to evaluate their potential against *Staphylococcus aureus* resistant to drugs, as well as to investigate the theoretical potential of these compounds to become new drug candidates through *in silico* studies involving Lipinski’s Rule of Five, topological polar surface area (TPSA), absorption percentage (% ABS), drug-likeness and drug score.

## 2. Results and Discussion

### 2.1. Chemistry

**Scheme 1:**
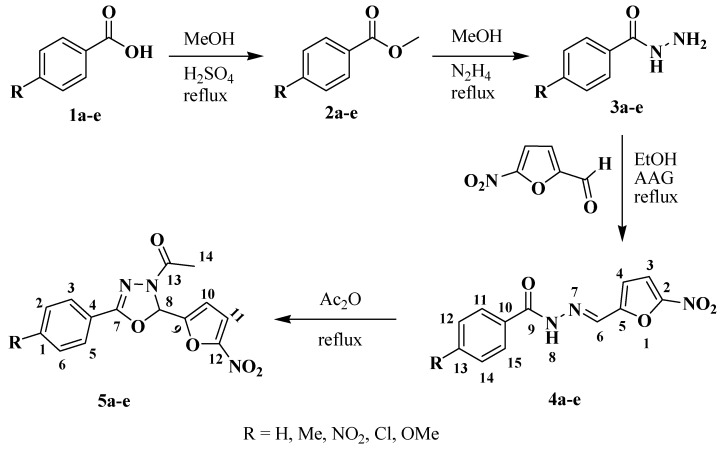
Synthetic route for the synthesis of the target molecules.

The synthesis of the new 1,3,4-oxadiazoline compounds **5a–e** was carried out according to procedures reported in the literature and the four synthetic stages are outlined in Scheme 1.

In step 1, the aromatic esters **2a–e** were obtained in quantitative yields by Fischer esterification of the respective aromatic carboxylic acids **1a–e**. In step 2, hydrazinolysis of the esters **2a–e** with hydrazine hydrate (80%) provided aroylhydrazides **3a–e** in excellent yields. Step 3 gave *N*-acylhydrazones **4a–e** in high yields and purity by condensation reaction between the respective aroylhydrazides **3a–e** and 5-nitro-2-furaldehyde in ethanol (95%) with glacial acetic acid as the catalyst [33].

Heterocyclic compounds of the 1,3,4-oxadiazoline class can be easily obtained by reacting *N*-acylhydrazones and acetic anhydride in excess with reflux [23,34,35]. The reaction of the *N*-acylhydrazones **4a–e** with acetic anhydride, Step 4, provided the compounds **5a–e** in moderate to good yields. The purity of compounds was verified examining their melting ranges and by gas chromatography. The structures of final and intermediate products were fully confirmed by IR, ^1^H-NMR and ^13^C-NMR spectroscopic techniques. The ^1^H-NMR, and ^13^C-NMR spectroscopy data was sufficient to confirm the formation of the 1,3,4-oxadiazole ring, as these compounds have very characteristic signals. Thus, in the ^1^H-NMR spectra of compounds **5a–e** two typical signals were observed, one assigned to the methyl protons (H-14) in the aliphatic region of (2.2 to 2.4 ppm) and another assigned to methinic protons (H-8) in the aromatic region (7.3 to 7.4 ppm), whereas for what was not observed, the typical signals for the NH (H-8), and CH=N (H-6) seen in the ^1^H-NMR spectra of compounds **4a–e** were no longer visible. In the ^13^C-NMR spectra, signals characteristic of C=O around 167 ppm, and alkyl around 23 ppm, as well as the oxadiazole ring signal (C-8) around 85 ppm and (C-7) about 153 ppm were observed, thus confirming the formation of the 1,3,4-oxadiazole ring. In the infrared spectrum, all the compounds **5a–e** showed amide C=O absorption bands around 1,670 cm^−1^, and C-O-C absorption stretches (oxadiazole ring) around 1,240 cm^−1^, and C=N absorptions (oxadiazole ring) at around 1,635 cm^−1^.

### 2.2. *In Silico* Study of Molecular Properties and Drug-Likeness

Molecular properties such as membrane permeability and oral bioavailability are usually associated with some basic molecular descriptors, such as log P (partition coefficient), molecular weight (MW), and the acceptors and donor for hydrogen bonding in a molecule. Using these molecular properties, Lipinski [36] established a controversial rule for drug design. Created in 1995 and published in 1997, it is known as the “Lipinski rule” or “rule of five”, it has this name, because each of the four parameters involved uses values that are multiples of five. The rule states that the compounds are more likely to be orally bioavailable if they obey the following criteria: log *p* ≤ 5, molecular weight ≤500, hydrogen bond acceptors ≤10, and hydrogen bond donors ≤5. Molecules that violate more than one of these rules may have problems with bioavailability. Therefore, this rule establishes some structural parameters relevant to the theoretical prediction of the oral bioavailability profile, and is widely used in designing new drugs. However, classes of compounds that are substrates for biological transporters such as antibiotics, antifungals, vitamins, and cardiac glycosides, are exceptions to the rule [36].

As such, we decided to carry out studies *in silico* for Lipinski parameters, as well as in the topological polar surface area (TPSA), the percentage of absorption (% ABS) and we include the drug-likeness and drug score (representing the combined physicochemical, pharmacokinetic and pharmacodynamic effects of a compound) for the *N*-acylhydrazone compounds **4a–e** and the 1,3,4-oxadiazoles **5a–e** in order to verify that these compounds exhibit good (theoretical) oral bioavailability potential.

The lipophilicity (log P) and topological polar surface area (TPSA) were calculated using the online software Molinspiration [37], while the aqueous solubility, drug-likeness and drug score were calculated using the OSIRIS property explorer software [38]. For the study of drug-likeness, the OSIRIS program uses a list of 5,300 molecular fragments, where the frequency of occurrence of each fragment is determined based on a collection of 3,300 drugs and 15,000 commercially available chemicals (Fluka) that are not drugs [38]. The percentage of absorption was estimated using the Equation: % ABS = 109 − (0.345 × TPSA), according to Zhao *et al*. [39]. The calculations data are shown in Table 1.

The calculation results show that all compounds meet the Lipinski rules of the five, suggesting that these compounds theoretically would not have problems with oral bioavailability. Generally, all compounds had scores of less than 5 for lipophilicity, ranging from 2.40 to 3.12 for the compounds **4a–e**, and from 1.91 to 2.40 for **5a–e** group. Most of the compounds also showed a PSA of less than 140 Å², (indicating a good permeability of the drug in the cellular plasma membrane), this, with the exception of compounds **4c** and **5c**, which showed higher values, The percentage of absorption (% ABS) calculated ranged from 50.46 to 74.35 for the **4a–e** group, and 50.61 to 74.20% for the **5a–e** group, see Table 1.

Most commercial products have LogS higher than −4.00 [30]. In Table 1, for the *N*-acylhydrazone compounds **4a–e**, LogS showed to be less than −4.00, ranging between −4.39 and −5.12, whereas for the 1,3,4-oxadiazole compounds **5a–e** showed LogS values in the range between −3.84 to −4.62.

Positive values for these drug scores indicate that the molecules contain predominantly pharmacophoric groups, which are often found in pharmaceuticals. A positive value for drug-likeness indicates that the compound contains predominantly fragments that are often present in most currently used drugs [38]. The drug score combines drug-likeness, lipophilicity, solubility, molecular weight and the risk of toxicity into a single numerical value that can be used to predict a global value for each compound as a potential new drug candidate [38].

The results in the calculations show that the compounds gave values for drug-likeness between −4.28 to 3.21, and −5.36 to 1.45 for **4a–e** and **5a–e**, respectively. All compounds showed positive values in the drug score calculation, the values ranged from 0.21 to 0.75, and from 0.3 to 0.52 for the compounds **4a–e** and **5a–e** respectively, see Table 1. The results show that these compounds, especially compounds **4a** and **5d**, have potential as new drug candidates.

### 2.3. Antibacterial Activity

The *N*-acylhydrazones **4a–e** [33] and the novel 1,3,4-oxadiazole compounds **5a–e** show effective antibacterial activity (MIC < 64 µg/mL [40]) for the various strains of *S. aureus* tested, with the MIC values between 4 μg/mL and 32 μg/mL Table 2. The ratios of MBC/MIC were 1 or 2, indicating that the anti-*S. aureus* effect of the compounds were bactericidal in nature (and not bacteriostatic) [41] (Table 2).

In general, for all strains tested, the majority of 1,3,4-oxadiazole compounds **5a–e** were less active than their precursors **4a–e** except for some compounds. For example, the compounds **5b–d** showed activity equal to the compounds **4b–d** for the strain (SA-1199B), while the compound **5c** was equipotent to the compound **4c** for the strains RN-4220, IS-58 and 05H. These results suggest that the introduction of the nucleus 1,3,4-oxadiazole between the two aromatic rings in the derivatives **4a–e** was not as effective in obtaining of compounds more active than the original *N*-acylhydrazones.

Regarding the effluxing strains (SA-1199B, RN-4220, IS-58), the highest activity was exhibited by compounds **4a**, **4b**, **4d**, **5b** and **5d**. Regarding the MARSA strains (007 and 05H) the most active compounds were **4b**, **4d**, **4e** and **5b**.

The biological activity exhibited by the compounds **4a–e** and **5a–e** shows a certain relationship with the lipophilicity of the molecules. Indeed, it may be noted that in most cases there was a decrease in activity of the compounds when the lipophilicity decreases. For example, when passing from the series **4a–e** to the series **5a–e** there is a reduction in lipophilicity of the compounds (Table 1), with a concurrent decrease in biological activity (Table 2). Indeed, considering all strains tested, the more lipophilic compounds **4b** and **4d** showed the best results of anti-staphylococcal activity.

**Figure 2:**
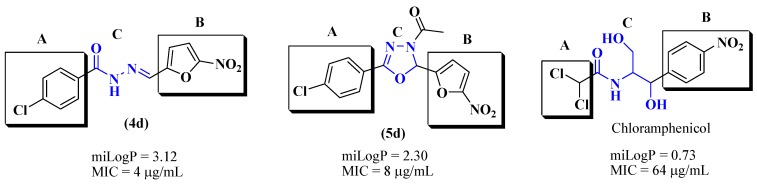
Structural similarity of chloramphenicol with the compounds **4d** and **5d**.

Coincidentally, the drug chloramphenicol chosen as standard in the studies of anti-staphylococcal activity has some structural similarity to the compounds **4a–e** and **5a–e**. In fact, this similarity is clearer considering the structures of the compounds **4d**, **5d** and chloramphenicol and taking into account three distinct regions in their structures: the region (A) containing the groups 4-chlorophenyl in the compounds **4d** and **5d** and dichloromethylene in the structure of the chloramphenicol, the region (B) containing the groups 4-nitrofuranyl in the compounds **4d** and **5d** and the 4-nitrophenyl in the structure in the chloramphenicol and the region (C) represented in blue, containing both amide functions (Figure 2).

Interestingly, all compounds were more active than chloramphenicol, and for strain MARSA (05H) the compound **4d** was 16-fold more potent than the standard. This difference in activity is directly related to the lipophilicity for compounds **4d**, **5d** and chloramphenicol (Figure 2). The increase in polarity in the region (**A**) appears to contribute in a significant way to reduce the biological activity, as shown for compounds **4d** and **4c**. Thus, in the structures of the compounds **4a–e** and **5a–e**, each of which have the same basic skeleton, the difference in activity of these compounds is related to the donor groups and electron withdrawing linked in the *para* position of benzene ring, thus providing different lipophilicity to these compounds.

However, because of these promising results, an extension of the series of the compounds analogues **4a–e** and **5a–e** is under development in order to carry out quantitative structure-activity relationship studies and thus have a better understanding on the relationship between the physicochemical properties and biological activity observed for these compounds.

## 3. Experimental

### 3.1. Chemistry

All used reagents and solvents were purchased from commercial sources (Sigma-Aldrich, Brazil) and used without a further purification. The progress of the reactions was monitored by thin layer chromatography (TLC) on silica gel plates. The purification of the compounds was performed by re-crystallization in ethanol and confirmed by determining the melting range on a MQAPF-3 brand hotplate, and by means of gas chromatography with low resolution mass spectrometry (GCMS-QP2010) Shimadzu. The spectra (IR) were obtained on a Shimadzu model IRPrestige-21 FTIR spectrometer, using KBr pellets. ^1^H-NMR and ^13^C-NMR spectra were obtained on two different machines: a Varian 200 NMR (200 MHz for ^1^H and 50 MHz for ^13^C) and a Varian 500 NMR (500 MHz and 125 MHz for ^1^H and ^13^C, respectively), Deuterated dimethyl sulfoxide (DMSO-*d_6_*) was used as solvent, and tetramethylsilane (TMS) as the internal standard. Chemical shifts (δ) were measured in units of parts per million (ppm) and coupling constants (*J*) in Hertz (Hz).

### 3.2. General Procedure for the Preparation of N-Acylhydrazones of ***4a–e***

A mixture of aroylhydrazides **3a–e** (3.0 mmol), 5-nitro-2-furaldehyde (0.423 g 3.0 mmol), absolute ethanol (30.0 mL) and glacial acetic acid (6 drops) was heated under reflux for 3 h. Then the reaction mixture was cooled to room temperature and poured into ice water. The precipitate formed was filtered, washed with water and ethanol, and purified by ethanol recrystallization technique.

*N′-((5-nitrofuran-2-yl)methylene)benzohydrazide* (**4a**). ^1^H-NMR (200 MHz, δ ppm, DMSO-*d_6_*): 7.23 (d, 1H, *J* = 3.84, Hz, H-3), 7.76 (d, 1H, *J* = 3.84 Hz, H-4), 8.0–7.48 (m, 5H, ArH), 8.38 (s, 1H, H-6), 12.22 (s, br, 1H, NH).

*4-Methyl-N′-((5-nitrofuran-2-yl)methylene)benzohydrazide* (**4b**). ^1^H-NMR (200 MHz, δ ppm, DMSO-*d_6_*): 2.37 (s, 3H, CH_3_).7.90–7.20 (m, 6H, aromatic and furan), 8.39 (s, 1H, H-6), 12.15 (s, br, 1H, NH).

*4-Nitro-N′-((5-nitrofuran-2-yl)methylene)benzohydrazide* (**4c**). ^1^H-NMR (200 MHz, δ ppm, DMSO-*d_6_*): 7.30 (d, 1H, *J* = 3.6, Hz, H-3), 7.79 (d, 1H, *J* = 3.6 Hz, H-4), 8.14 (d, 2H, *J* = 9.0 Hz, H-12,14), 8.41 (d, 2H, *J* = 9.0 Hz, H-11,15), 8.41 (s, 1H, H-6), 12.48 (s, br, 1H, NH).

*4-Chloro-N′-((5-nitrofuran-2-yl)methylene)benzohydrazide* (**4d**). ^1^H-NMR (200 MHz, δ ppm, DMSO-*d_6_*): 7.26 (d, 1H, *J* = 3.96, Hz, H-3), 7.59 (d, 2H, *J* = 8.58 Hz, H-12,14), 7.78 (d, 1H, *J* = 3.9 Hz, H-4), 7.95 (d, 2H, *J* = 8.64 Hz, H-11,15), 8.38 (s, 1H, H-6), 12.26 (s, br, 1H, NH).

*4-Methoxy-N′-((5-nitrofuran-2-yl)methylene)benzohydrazide* (**4e**) ^1^H-NMR (200 MHz, δ ppm, DMSO-*d_6_*): 3.83 (s, 3H, OCH_3_), 7.06 (d, 2H, *J* = 9.48 Hz, H-12,14), 7.23 (d, 1H, *J* = 3.90, Hz, H-3), 7.77 (d, 1H, *J* = 3.9 Hz, H-4), 7.91 (d, 2H, *J* = 8.82 Hz, H-11,15), 8.38 (s, 1H, H-6), 12.12 (s, br, 1H, NH).

### 3.3. General Procedure for the Preparation of 1-(2-(5-Nitrofuran-2-yl)-5-(4-substituted-phenyl)-1,3,4-oxadiazol-3(2H)-yl)ethanones ***5a–e***

A mixture of *N*-acylhydrazones **4a–e** (2.0 mmol) and acetic anhydride in excess (6.0 to 15.0 mL) was heated under reflux at a temperature of 140 °C for 1 to 2.5 h. The reaction mixture was then cooled to a temperature of 80–100 °C and poured into ice water (30.0 mL) to decompose the excess acetic anhydride. The reaction mixture was stirred vigorously until a precipitate formed, which was filtered, washed twice with aqueous NaHCO_3_ (5.0%), and then with water. The purification of the compounds was performed by recrystallization from ethanol, or ethanol/water.

*1-(2-(5-Nitrofuran-2-yl)-5-phenyl-1,3,4-oxadiazol-3(2H)-yl)ethanone* (**5a**). Yield: 66.4%; m.p. 178–180 °C; ^1^H-NMR (500 MHz, DMSO-*d_6_*) δ (ppm): 2.28 (s, 3H, H-14), 7.21 (d, 1H, *J* = 3.5Hz), 7.37 (s, 1H, H-8), 7.54 (t, 2H, *J* = 7.5 Hz), 7.61 (t, 1H, *J* = 7.5 Hz, H-1), 7.69 (d, 1H, *J* = 3.5 Hz, H-11), 7.84 (d, 2H, *J* = 7.5 Hz, H-3,5); ^13^C-NMR (125 MHz, DMSO-*d_6_*) δ (ppm): 21.04 (C-14), 84.34 (C-8), 113.2 (C-10), 114.54 (C-11), 123.31 (C-4), 126.57 (C-3,5), 129.12 (C-2,6), 132.15 (C-1), (C-9, no), 150.46 (C-12), 154.51 (C-7), 167.18 (C-13); IR (KBr): 1,670 (C=O), 1,631 (C=N), 1,242 (C-O-C), 1,535 and 1,361 (NO_2_), 1,593 and 1,500 (C=C) cm^−1^.

*1-(2-(5-Nitrofuran-2-yl)-5-(methylphenyl)-1,3,4-oxadiazol-3(2H)-yl)ethanone* (**5b**). Yield: 45.9%; m.p. 160–162 °C; ^1^H-NMR (500 MHz, DMSO-*d_6_*) δ (ppm): 2.28 (s, 3H, H-14), 2.38 (s, 3H, CH_3_), 7.85–7.18 (m, 7H); ^13^C-NMR (125 MHz, DMSO-*d_6_*) δ (ppm): 21.53 (CH_3_), 21.56 (C-14), 84.58 (C-8), 113.61 (C-10), 114.80 (C-11), 120.93 (C-4), 126.10 (C-3,5), 130.43 (C-2,6), 142.84 (C-1), 150.47 (C-9), 150.95 (C-12), 155.04 (C-7), 167.49 (C-13); IR (KBr): 1,674 (C=O), 1,635 (C=N), 1,238 (C-O-C), 1,535 and 1,354 (NO_2_), 1,593 and 1,508 (C=C) cm^−1^.

*1-(2-(5-Nitrofuran-2-yl)-5-(4-nitrophenyl)-1,3,4-oxadiazol-3(2H)-yl)ethanone* (**5c**). Yield: 50.6%; m.p. 188–190 °C; ^1^H-NMR (500 MHz, DMSO-*d_6_*) δ (ppm): 2.31 (s, 3H, H-14), 7.26 (d, 1H, *J* = 3.5 Hz), 7.43 (s, 1H, H-8), 7.70 (d, 1H, *J* = 4.0 Hz, H-11), 8.06 (d, 2H, *J* = 8.5 Hz, H-3,5), 8.34 (d, 2H, *J* = 9.0 Hz, H-2,6); ^13^C-NMR (125 MHz, DMSO-*d_6_*) δ (ppm): 20.98 (C-14), 85.09 (C-8), 113.02 (C-10), 114.66 (C-11), 124.30 (C-2,6), 127.78 (C-3,5), 129.09 (C-4), 149.1 (C-1), 150.00 (C-12), 151.72 (C-9), 152.94 (C-7), 167.39 (C-13); IR (KBr): 1,670 (C=O), 1,627 (C=N), 1,246 (C-O-C), 1,543 and 1,354 (NO_2_), 1,597 and 1,516 (C=C) cm^−1^.

*1-(2-(5-Nitrofuran-2-yl)-5-(chlorophenyl)-1,3,4-oxadiazol-3(2H)-yl)ethanone* (**5d**). Yield: 74.3%); m.p. 188–190 °C; ^1^H-NMR (500 MHz, DMSO-*d_6_*) δ (ppm): 2.28 (s, 3H, CH_3_), 7.23 (d, 1H, *J* = 4.0 Hz, H-10), 7.39 (s, 1H, H-8), 7.61 (d, 2H, *J* = 9.00 Hz, H-2,6), 7.70 (d, 1H, *J* = 4.0 Hz, H-11), 7.84 (d, 2H, *J* = 8.50 Hz, H-3,5); ^13^C-NMR (125 MHz, DMSO-*d_6_*) δ (ppm): 20.98 (C-14), 84.59 (C-8), 113.07 (C-10), 114.47 (C-11), 122.17 (C-4), 128.30 (C-3,5), 129.27 (C-2,6), 136.79 (C-1), 150.26 (C-12), (C-9, no), 153.66 (C-7), 167.16 (C-13); IR (KBr): 1,670 (C=O), 1,631 (C=N), 1,242 (C-O-C), 1,535 and 1,357 (NO_2_), 1,593 and 1,500 (C=C) cm^−1^.

*1-(2-(5-Nitrofuran-2-yl)-5-(methoxyphenyl)-1,3,4-oxadiazol-3(2H)-yl)ethanone* (**5e**). Yield: 40.8%; m.p. 181–183 °C; ^1^H-NMR (200 MHz, DMSO-*d_6_*) δ (ppm): 2.27 (s, 3H, CH_3_), 3.84 (s, 3H, OCH_3_), 7.08 (d, 2H, *J* = 8.64 Hz, H-2,6), 7.20 (d, 1H, *J* = 3.90 Hz, H-10), 7.34 (s, 1H, H-8), 7.70 (d, 1H, *J* = 3.54 Hz, H-11), 7.79 (d, 2H, *J* = 8.82 Hz, H-3,5); ^13^C-NMR (50 MHz, DMSO-*d_6_*) δ (ppm): 21.49 (C-14), 55.89 (OCH_3_), 84.42 (C-8), 113.61 (C-10), 114.82 (C-11), 114.97 (C-2,6), 115.81 (C-4), 128.93 (C-3,5), 151.03 (C-12), 152.16 (C-9), 154.94 (C-7), 162.59 (C-1), 167.37 (C-13); IR (KBr): 1,662 (C=O), 1,635 (C=N), 1,257 (C-O-C), 1,535 and 1,357 (NO_2_), 1,608 and 1,504 (C=C) cm^−1^.

### 3.4. Bacterial Strains

The strains of *S. aureus* used were: SA-1199B, which overexpresses the NorA gene encoding the NorA efflux protein for fluoroquinolones (and other drugs) [42]; RN4220, harbouring plasmid pUL5054, which carries the gene encoding the MsrA macrolide efflux protein [43]; IS-58, which possesses the TetK tetracycline efflux protein [44], and two clinical strains (007 and 005H), resistant to amino glycosides and to methicillin (MARSA). The effluxing strains were kindly provided by Professor Simon Gibbons (University of London, London, UK), and the MARSA strains were obtained from the culture collection of the Laboratory of Microorganism Genetics (Department of Molecular Biology, Federal University of Paraíba). All strains were maintained on blood agar base slants (BAB, Difco Laboratories Ltd., Brazil), and prior to use, the cells were grown overnight at 37 °C in brain heart infusion broth (BHI, Difco Laboratories Ltd., Detroit, MI, USA).

### 3.5. Antibacterial Activity

The stock solutions of the compounds were prepared in DMSO which at its highest final concentration after dilution in the broth (4%) caused no inhibition of bacterial growth. The minimum inhibitory concentrations (MICs) of the compounds were determined in BHI by microdilution assay using a suspension of ca. 105 cfu/mL and a drug concentration range of 256 to 0.5 μg/mL (two-fold serial dilutions). The MIC is defined as the lowest concentration at which no growth is observed. The dye resazurin was used for better visualization of the bacterial growth. Subcultures were made on BAB from the last well that showed visible growth, and from all successive wells. The minimum bactericidal concentration (MBC) is defined as the lowest drug concentration that gives no growth on the agar.

## 4. Conclusions

We have described the synthesis and evaluation of anti-staphylococcal activity for *N*-acylhydrazones **4a–e**, and five new 1,3,4-oxadiazole compounds **5a–e** against several strains of *Staphylococcus aureus*. All compounds were fully characterized using IR, ^1^H- and ^13^C-NMR spectroscopic techniques. In studies of anti-staphylococcal activity, all the compounds exhibited good results with MIC between 4 μg/mL and 32 μg/mL, being more potent than the standard drug chloramphenicol. *In silico* studies, all compounds had a desirable profile to be new drug candidates.

## Figures and Tables

**Table 1:** Calculated absorption (% ABS), polar surface area (PSA), LogS, Lipinski parameters, drug-likeness and drug score of the compounds **4a–e** and **5a–e**.

Comp.	Lipinski’s Parameters					TPSA (Å²)	% ABS	Log S	Drug-likeness	Drug score
	HBA	HBD	MW	miLogP	Violations					
**4a**	7	1	259.22	2.44	0	100.43	74.35	−4.39	3.21	0.75
**4b**	7	1	273.27	2.89	0	100.43	74.35	−4.73	1.76	0.66
**4c**	10	1	304.24	2.40	0	146.25	50.46	−4.85	−4.04	0.21
**4d**	7	1	293.68	3.12	0	100.43	74.35	−5.12	4.28	0.64
**4e**	8	1	289.27	2.50	0	109.66	71.17	−4.41	2.96	0.74
**5a**	8	0	301.25	1.95	0	100.87	74.20	−3.89	0.02	0.50
**5b**	8	0	315.28	2.40	0	100.87	74.20	−4.23	−1.56	0.37
**5c**	11	0	346.25	1.91	1	146.70	50.61	−4.35	−5.36	0.30
**5d**	8	0	335.68	2.30	0	100.87	74.20	−4.62	1.45	0.52
**5e**	9	0	331.28	2.01	0	110.11	71.01	−3.90	−2.77	0.34

**Table 2:** Minimum Inhibitory Concentration (MIC) and Minimum Bactericidal Concentration (MBC) of *N*-acylhydrazone compounds **4a–e** and 1,3,4-oxadiazoles **5a–e** against strains of *S. aureus*.

Compound	Strains				
	SA-1199B	RN-4220	IS-58	007	05H
**4a**	16-16 *	8-8	8-8	8-8	8-8
**4b**	16-32	8-8	8-8	8-8	4-4
**4c**	32-32	16-32	16-16	8-16	8-8
**4d**	16-16	8-8	8-16	8-8	4-4
**4e**	16-16	16-16	8-8	8-8	4-4
**5a**	32-32	16-32	16-16	32-32	16-16
**5b**	16-32	16-32	16-16	8-8	8-8
**5c**	32-32	16-32	16-16	16-16	8-8
**5d**	16-32	16-32	16-16	16-16	8-8
**5e**	32-32	32-32	32-32	16-16	8-8
**Chloramphenicol**	64-64	64-128	64-128	64-128	64-128

* MIC-MBC (µg/mL).
